# Development and Exploratory Evaluation of the Dietary Plastic Exposure Score for Mexican Populations in University Students

**DOI:** 10.3390/nu18142242

**Published:** 2026-07-09

**Authors:** Alejandro Lopez-Moro, Javier Conde-Pipó, Miriam Aracely Anaya-Loyola, Miguel Mariscal-Arcas

**Affiliations:** 1 Health Science and Nutrition Research (HSNR-CTS1118), Department of Nutrition and Food Science, School of Pharmacy, University of Granada, 18071 Granada, Spain; alexlopez@ugr.es; 2 Department of Health Sciences, Faculty of Health Sciences, University of Jaén, 23009 Jaén, Spain; 3 School of Natural Science, Autonomous University of Quétaro, Quétaro 76230, Mexico; aracely.anaya@uaq.mx; 4 Instituto de Investigación Biosanitaria de Granada (ibs.GRANADA), 18012 Granada, Spain

**Keywords:** plastic exposure, dietary patterns, ultra-processed foods, Mexico, food packaging

## Abstract

**Background:** Plastic food-contact materials are increasingly recognised as a potential source of dietary exposure to endocrine-disrupting chemicals, particularly through packaged, canned, and ultra-processed foods. However, culturally adapted tools for estimating long-term exposure-related dietary behaviours in Latin American populations remain limited. This study aimed to develop and provide a preliminary evaluation of the Dietary Plastic Exposure Score for Mexican Populations (DPES-MX), and to characterise exposure-related dietary patterns in Mexican university students. **Methods:** A cross-sectional study was conducted in 152 university students from Querétaro, Mexico. Anthropometric, lifestyle, and dietary information were collected using standardised questionnaires and a culturally adapted food frequency questionnaire. The DPES-MX incorporated dietary and food-handling practices linked to potential migration of plastic-related compounds from food-contact materials, including canned foods, packaged beverages, takeaway foods, microwave heating in plastic containers, and convenience-oriented eating behaviours. Principal component analysis (PCA) and adjusted linear regression models were applied to explore associations between dietary patterns and DPES-MX scores. **Results:** Men showed slightly higher DPES-MX scores than women, although differences were not statistically significant. Women were more frequently classified within the low DPES-MX score category, and female sex was independently associated with lower DPES-MX scores in adjusted models. PCA identified a dietary component characterised by alcoholic beverages, snacks, sweets, sugar-sweetened beverages, and processed foods that was positively associated with higher DPES-MX scores (β = 0.81, *p* < 0.001). **Conclusions:** The DPES-MX could serve as a culturally adapted epidemiological tool for identifying dietary and behavioural practices linked to potential exposure to plastic-related food-contact materials in Mexican populations. Convenience-oriented dietary patterns appeared to be associated with higher DPES-MX scores.

## 1. Introduction

Plastic food-contact materials are extensively used in modern food systems because they facilitate food preservation, storage, transport, and commercialization on a large scale [1]. Nevertheless, these materials can act as a source of chemical contamination, since compounds present in packaging can migrate into foods through diffusion-dependent processes, including bisphenols, phthalates, styrene derivatives, and other endocrine-disrupting chemicals (EDCs), several of which remain incompletely characterised from a toxicological perspective [2]. Recent reviews have emphasised that chemical transfer from food-contact materials is governed by interacting permeation, migration, and sorption processes, and may occur through several mechanisms, including direct-contact migration, gas-phase migration, penetration or diffusion migration, set-off migration, and condensation/distillation migration [3]. In addition to low-molecular-weight chemicals, food-contact articles have also been identified as potential sources of micro- and nanoplastics released into foods or food simulants during normal or intended use [4].

Migration processes are influenced by factors such as contact time, temperature, microwave heating, and the physicochemical characteristics of foods, particularly fatty and acidic matrices, supporting the role of packaging as an important source of dietary exposure to food-contact chemicals (FCC) [5]. In fact, dietary intake is considered one of the main routes of human exposure to bisphenol A (BPA), particularly through packaged and processed foods [6]. Higher urinary BPA concentrations have been associated with the consumption of canned foods, beverages stored in plastic or metallic containers, and microwave-heated meals [7,8].

Plastic-associated chemicals can interfere with endocrine regulation and have been linked to a higher risk of metabolic syndrome, cardiovascular disorders, obesity, infertility or neurodevelopmental outcomes, although causal relationships in humans remain difficult to establish due to methodological heterogeneity and variability in exposure assessment [5,6].

Despite increasing interest in dietary exposure to plastic-related compounds, exposure assessment remains challenging. Biomonitoring and detailed dietary assessment methods may improve exposure estimation; however, they are costly, mainly reflect short-term exposure, and require substantial participant burden and specialised personnel [7]. Consequently, simplified questionnaires and exposure scores have been developed to estimate plastic-related dietary exposure based on eating behaviours associated with canned foods, packaged products, and heating practices involving plastic containers [5,7,8,9].

Latin America, a region comprising 33 countries and approximately 650 million inhabitants, is undergoing a rapid nutritional transition characterised by rising obesity rates and increasing consumption of processed foods, making it highly relevant for food environment and dietary exposure research [10]. Within this context, Mexico represents a particularly relevant setting. Although the country preserves a rich culinary tradition rooted in pre-Hispanic staple foods such as maize, beans, chilli, tomato, and squash, rapid urbanisation, economic globalisation, and major sociocultural transformations have progressively modified traditional eating habits, favouring more Westernised dietary patterns characterised by greater consumption of processed, packaged, energy-dense, and highly industrialised foods, together with one of the highest obesity prevalences among high-income countries [11,12].

In addition, eating outside the home and the widespread availability of street foods, takeaway meals, and industrialised food products are deeply integrated into contemporary Mexican food environments, potentially increasing exposure to food-contact materials associated with packaging and food storage practices [12,13]. This phenomenon has occurred alongside progressive changes in the retail food environment, characterised by progressive displacement of traditional fruit and vegetable stores and increasing availability of convenience stores, fast-food outlets, and large supermarket chains offering energy-dense and ultra-processed products [14]. This context may be particularly relevant considering the frequent consumption of canned foods such as tuna and jalapeño peppers in the Mexican population, together with the current absence of specific legislation regulating food-contact materials in the country [15].

At the same time, Mexican university populations have shown dietary patterns characterised by low intake of plant-based foods together with frequent consumption of sugary drinks and industrialised products [12]. Such eating behaviours may favour greater exposure to FCC due to increased reliance on packaged, processed, and ready-to-consume foods.

Although biomonitoring methods may improve exposure assessment, their economic and logistical limitations reduce their applicability in large epidemiological studies. In this context, simplified questionnaire-based approaches may represent useful tools for identifying behavioural patterns and food-related practices potentially associated with greater exposure to plastic-related food-contact (PRFC) compounds [8]. Existing instruments have mainly been developed in non-Latin American populations and may not fully capture specific characteristics of contemporary Mexican food environments, including street-food consumption, takeaway practices, canned products, and convenience-based eating behaviours. Several questionnaire-based or survey-derived approaches have previously been proposed to estimate dietary exposure to plastic-related food-contact chemicals, particularly BPA. Nomura et al. [8] developed a questionnaire focused on known dietary and non-dietary sources of BPA exposure, while Hartle et al. [7] proposed a dietary BPA exposure score based on foods and practices commonly related to BPA migration, including canned foods, packaged foods, and microwave-related behaviours. More recently, Harray et al. [6] developed the Dietary Plastics Score using a 24 h dietary recall framework to capture contact with plastic packaging during food intake, and Patti et al. [5] tested a diet-based BPA score for epidemiological research on child neurodevelopment. These tools provide important precedents for simplified exposure assessment, but most were developed in non-Latin American settings and may not fully capture food practices common in Mexican university environments, such as street-food consumption, takeaway meals, bottled beverages, canned foods, and hot foods served in disposable containers.

Therefore, the present study aimed to develop and evaluate a culturally adapted dietary and behavioural exposure score for the epidemiological assessment of long-term exposure to PRFC materials in Mexican populations, as well as to characterise exposure-related dietary and behavioural patterns among Mexican university students.

## 2. Materials and Methods

### 2.1. Study Design and Participants

This cross-sectional, descriptive, and comparative study was conducted between January 2025 and June 2025 on university students from Querétaro, Mexico. The study protocol followed the principles established in the Declaration of Helsinki, and all participants provided written informed consent prior to participation. The study protocol was approved by the Research Ethics Committee of the Andalusian Public Health Service and the University of Murcia (protocol code: ID-1248/2016; date of approval: 15 March 2016).

Participants were recruited through classroom announcements and institutional dissemination channels. A total of 350 students were initially invited to participate. Interested students received detailed information about the aims and procedures of the study, after which 206 agreed to continue and provided written informed consent. Of these, 23 students were excluded because they did not meet the eligibility criteria or reported conditions potentially affecting dietary habits. A further 31 participants were excluded because of incomplete dietary information, incomplete DPES-MX data, or implausible questionnaire responses. The final analytical sample therefore included 152 students.

Inclusion criteria were: (a) being enrolled as an active university student, (b) being between 18 and 30 years of age, and (c) providing complete questionnaire data. Participants with incomplete dietary information, implausible nutritional records, diagnosed eating disorders, or chronic diseases potentially affecting dietary habits were excluded from the final analyses.

No formal a priori sample-size calculation was performed because this was an exploratory, evaluation study aimed at developing and describing the DPES-MX in a university population. The final sample size was determined by the number of eligible participants with complete data during the recruitment period.

### 2.2. Anthropometric Assessment

Anthropometric measurements were obtained by previously trained personnel following standardised procedures. Height was recorded in centimetres using a wall-mounted stadiometer (Seca 214, SECA Deutschland, Hamburg, Germany), while body weight was measured in kilograms with a high-precision digital scale (Tanita BC-418, Tanita, Tokyo, Japan). Participants were assessed barefoot and wearing light clothing, applying a 0.6 kg correction factor to account for clothing weight [16]. Body mass index (BMI) was subsequently calculated as body weight divided by height squared (kg/m^2^).

### 2.3. Sociodemographic, Lifestyle, and Physical Activity Assessment

An ad hoc questionnaire was developed to collect sociodemographic, lifestyle, and eating-behaviour information. Participants reported sex, age, smoking habits, alcohol consumption, self-perceived stress, and habitual food-related behaviours, including frequency of home cooking, current dieting status, food purchasing priorities (quality, price, or both), self-perceived nutrition knowledge, and calorie-counting practices [12]. Self-perceived stress was assessed using a dichotomous yes/no item and was coded as a categorical variable for the regression analyses.

Physical activity was assessed using the Rapid Assessment of Physical Activity Questionnaire (RAPA-Q), a validated instrument frequently employed in adult populations to classify physical activity levels according to aerobic and strength-related activities. Participants were categorised as sedentary, active, or very active according to questionnaire scoring criteria [17].

### 2.4. Dietary Assessment

Dietary intake and habitual food consumption were evaluated using a semi-quantitative food frequency questionnaire (FFQ) previously validated in Mexican adolescent and adult populations [12,14,18]. This instrument was based on the semi-quantitative FFQ used in the Mexican National Health and Nutrition Survey, which includes 140 food items consumed during the seven days prior to the interview, with predefined frequency and portion-size categories. The original validation study reported moderate validity for energy, macronutrients, and micronutrients, as well as good ability to rank individuals according to dietary intake when compared with repeated 24 h dietary recalls [14].

The food list and terminology were reviewed with local Mexican researchers before data collection to ensure regional adequacy for the Querétaro university context. This regional review focused on the wording of locally used food names, commonly consumed preparations, beverages, snacks, condiments, and packaged or convenience foods relevant to the study objectives.

Before final data collection, a preliminary field check was performed using 24 h dietary recalls in a subsample of 20 participants to identify potentially missing foods or locally relevant items not adequately captured by the FFQ. This procedure was used to check the contextual adequacy of the food list for the Querétaro setting, including regionally relevant preparations such as gorditas de migajas, but it was not intended as an additional validation study.

Food items from the FFQ were converted into estimated intake values and then grouped into nutritionally and culturally coherent food categories before statistical analysis [12]. The final food groups included dairy products, red meat, poultry, processed meat products, eggs, fish and seafood, vegetables, fruits, legumes and soy, tubers, cereals and grain-based foods, fats and oils, sugars and sweets, cookies and snacks, sugar-sweetened beverages, sugar-free beverages, alcoholic beverages, nuts, and sauces and condiments. The term ultra-processed foods was used according to the NOVA classification, which defines ultra-processed foods as industrial formulations of ingredients, often containing additives and substances not commonly used in home cooking, and typically designed to be ready-to-eat, ready-to-drink, or ready-to-heat [19]. In the present study, no separate NOVA-based ultra-processed food score was calculated. Instead, FFQ-derived food groups commonly considered compatible with ultra-processed or convenience-oriented products, such as cookies and snacks, sugars and sweets, sugar-sweetened beverages, packaged beverages, instant noodles, and microwavable packaged foods, were used within the descriptive and PCA-based analyses. These food-group variables were used for descriptive analyses and as input variables for the principal component analysis.

### 2.5. Development of the DPES-MX

The DPES-MX was based on questionnaire items asking participants about usual food wrapping; frequency of takeaway or fast-food consumption according to packaging type; weekly intake of canned foods; weekly intake of microwavable packaged foods; usual packaging for juice, water, and soft drinks; consumption of foods from flexible plastic containers or pouches; microwave heating in plastic containers; storage of hot food in plastic containers; and use of plastic water dispensers. The source of each DPES-MX item was defined as follows. Food-frequency items, including canned foods and microwavable packaged foods, were derived from the semi-quantitative FFQ. Packaging-choice and food-handling items, including usual food wrapping, takeaway or fast-food packaging type, beverage packaging, foods consumed from flexible plastic containers or pouches, microwave heating in plastic containers, storage of hot food in plastic containers, and use of plastic water dispensers, were collected through the ad hoc sociodemographic, lifestyle, and eating-behaviour questionnaire.

The selection and weighting of these items were informed by previous dietary BPA and plastic-exposure questionnaires and by evidence on migration from food-contact materials. Variables were coded using binary or ordinal categories according to exposure frequency and were weighted a priori according to the expected migration potential linked to each practice. Frequency-based items were coded using a common weekly frequency scale: 0 = never, 1 = 1–2 times/week, 2 = 3–4 times/week, and 3 = 5–7 times/week. Packaging-choice variables were coded as binary indicators, with 1 assigned when the participant selected the specific packaging material and 0 when another non-missing option was selected.

The weighting scheme was defined as a relative, theory-driven hierarchy, based on previous dietary BPA and plastic-exposure scoring approaches [5,6,7,8]. Previous BPA exposure scores have assigned the highest relative weight to canned foods, whereas packaged foods, microwavable foods, canned beverages, and restaurant meals have received lower weights because of their lower or less-consistent BPA content [7,8]. Experimental and dietary exposure studies also support the relevance of canned foods, beverage packaging, and microwave containers as sources of BPA migration, with migration influenced by factors such as temperature, heating time, food matrix, and container type [20,21]. A weight of 1.00 was assigned to canned foods and to behaviours involving microwave heating or storage of hot food in plastic containers. A weight of 0.75 was assigned to direct-contact plastic or polystyrene takeaway packaging and canned beverages. A weight of 0.50 was assigned to intermediate exposure sources, including coated or mixed packaging, microwavable packaged foods, flexible plastic packaging, plastic beverage containers, and plastic water dispensers. A weight of 0.25 was assigned to lower-intensity or indirect packaging-related exposures, such as carton packaging or paper wrapping. Each item score was multiplied by its predefined weight, and the total DPES-MX score was calculated as the sum of all weighted item scores. The index formula was: DPES-MX = Σ(Ci × Wi), where Ci represents the coded value of item i and Wi represents the predefined theoretical weight assigned to that item. The theoretical total DPES-MX score ranged from 0 to 36.25 points. Higher scores indicate a greater frequency of dietary and food-handling practices involving plastic-related food-contact materials. Participants were subsequently categorised into low, moderate, and high DPES-MX score categories according to tertiles of the total DPES-MX score distribution in the overall sample.

The DPES-MX was conceived as a formative exposure-oriented index rather than as a reflective psychometric scale. Therefore, its items were not expected to be interchangeable manifestations of a single latent construct, but complementary indicators of different dietary and food-handling practices that may contribute to contact with plastic-related food-contact materials. The coding system and weighting structure of the DPES-MX are summarised in Table 1.

### 2.6. Statistical Analysis

Statistical analyses were performed using R statistical software, version 2026.01.2+418, (R Foundation for Statistical Computing, Vienna, Austria). Continuous variables were expressed as means and standard deviations, whereas categorical variables were presented as frequencies and percentages. Participants with incomplete dietary information, implausible nutritional records, diagnosed eating disorders, or chronic diseases potentially affecting dietary habits were excluded from the final analytical sample. No data imputation was performed. Descriptive analyses were conducted using available data for each variable, whereas regression models were fitted using complete cases for the variables included in each model. Group comparisons were performed using Student’s *t*-test or Mann–Whitney U test for continuous variables and chi-square or Fisher’s exact tests for categorical variables, as appropriate. Effect sizes were reported to complement *p*-values and to provide information on the magnitude of between-sex differences. Cohen’s d was used for continuous variables, and Cramer’s V was used for categorical variables.

Principal component analysis (PCA) was applied to standardised food-group intake variables derived from the FFQ in order to identify major food-consumption dimensions. Variables were centred and scaled before PCA. The suitability of the food-group matrix for PCA was assessed using the Kaiser–Meyer–Olkin measure of sampling adequacy and Bartlett’s test of sphericity. The food-group matrix showed excellent suitability for PCA (KMO = 0.937), and Bartlett’s test of sphericity was significant (χ^2^ = 1635, *df* = 136, *p* < 0.001). Components were retained according to a combination of eigenvalues greater than 1, explained variance, scree-plot inspection, and interpretability. The first three components were retained and together explained 64.9% of the total variance. Unrotated principal components were used because the objective was data reduction and the generation of orthogonal food-pattern scores for subsequent regression analyses, rather than the identification of correlated latent dietary constructs. Component scores for PC1, PC2, and PC3 were extracted and entered into adjusted linear regression models to explore their association with DPES-MX scores. In this study, PCA was used as a descriptive data-reduction procedure rather than as a method to define mutually exclusive food-group categories or latent dietary constructs. Therefore, component labels were assigned only to aid interpretation of the extracted scores. The PCA-derived scores were subsequently used as summary variables to explore whether broad food-consumption dimensions were associated with DPES-MX scores.

Adjusted linear regression models were used to explore associations between DPES-MX scores and PCA-derived food-pattern scores. Covariates were selected a priori based on their potential relationship with dietary behaviours, lifestyle patterns, and exposure-related food-contact practices. The final model included sex, age, BMI, physical activity level, smoking status, alcohol consumption, self-perceived stress, and the first three PCA-derived food-pattern scores. Reference categories were men for sex, active participants for physical activity, smokers for smoking status, non-drinkers for alcohol consumption, and participants without self-perceived stress for stress.

Model diagnostics were assessed by visual inspection of residual-versus-fitted plots, Q–Q plots, and scale-location plots. Potential influential observations were explored using Cook’s distance. Multicollinearity was assessed using variance inflation factors or generalised variance inflation factors for categorical predictors. Adjusted VIF/GVIF values below 5 were considered acceptable. Statistical significance was established at *p* < 0.05 for all analyses.

## 3. Results

Table 2 presents the anthropometric and lifestyle characteristics of the study population according to sex. The sample included 152 university students of whom 55.9% were men and 44.1% were women. No significant sex-related differences were observed for age (*p* = 0.233).

Men had significantly higher body weight, height, and BMI values than women (all *p* < 0.001). Specifically, men presented a mean BMI of 25.41 ± 6.05 kg/m^2^, whereas women showed a mean BMI of 22.82 ± 3.49 kg/m^2^. Regarding BMI classification, a higher prevalence of overweight and obesity was observed among men, although the overall distribution did not reach statistical significance (*p* = 0.071).

No statistically significant differences were identified between sexes for physical activity level, smoking habits, or alcohol consumption (all *p* > 0.05), although alcohol consumption tended to be more frequent among men (30.6% vs. 17.9%).

Regarding eating habits and nutrition-related behaviours, no significant sex-related differences were identified for cooking habits, dieting status, perceived nutritional knowledge, or self-reported healthy eating behaviours (all *p* > 0.05). Most participants reported usually cooking at home (56.6%) and considering both price and quality when selecting foods (68.4%). Calorie counting showed a trend toward higher prevalence among men compared with women (21.6% vs. 8.1%, *p* = 0.052).

DPES-MX domain scores and DPES-MX score categories according to sex are presented in Table 3. Overall, men showed slightly higher total DPES-MX scores compared with women (12.98 ± 2.96 vs. 12.12 ± 2.95), although the difference did not reach statistical significance (*p* = 0.089). Among the evaluated domains, significant sex-related differences were mainly identified in variables associated with microwavable packaged foods. Men reported higher consumption of microwavable packaged foods overall (0.50 ± 0.53 vs. 0.27 ± 0.29, *p* = 0.013), including instant noodles (0.38 ± 0.35 vs. 0.25 ± 0.28, *p* = 0.038) and microwave rice products (0.12 ± 0.30 vs. 0.01 ± 0.09, *p* = 0.006). No significant differences between sexes were observed for canned food exposure, heating food in plastic containers, beverage packaging exposure, or flexible plastic packaging habits (all *p* > 0.05).

When participants were categorised according to DPES-MX score tertiles, women showed a higher proportion in the low DPES-MX score category compared with men (41.8% vs. 25.9%, *p* = 0.037). No statistically significant sex-related differences were identified for moderate or high DPES-MX score categories.

No significant differences in food-group intake were observed across DPES-MX score categories (all *p* > 0.05). However, participants with higher DPES-MX scores tended to report greater consumption of ultra-processed foods, including cookies and snacks, sugar-sweetened beverages, and alcoholic beverages, whereas dairy and fruit intake tended to be lower.

The first three principal components explained 64.9% of the total variance in food-group intake, with PC1, PC2, and PC3 accounting for 52.4%, 6.1%, and 6.0% of the variance, respectively. PC1 showed loadings in the same general direction across most food groups and was therefore interpreted as a general dietary-intake dimension rather than as a specific animal-, plant-, or packaging-related pattern. PC2 captured a contrast characterised by higher loadings for alcoholic beverages, fish and seafood, sugars and sweets, cookies and snacks, and sugar-sweetened beverages, together with negative loadings for poultry and cereals, rice, and bread. Because several of the positive-loading food groups are commonly consumed as packaged, processed, or convenience products, PC2 was cautiously interpreted as a convenience/packaged-food-related dietary dimension rather than as a specific food-group category. PC3 showed higher positive loadings for nuts, legumes and soy, vegetables, and tubers, together with negative loadings for dairy products, poultry, and red meat, and was interpreted as a mixed plant-based/traditional-food dimension. Although PC1 explained the largest proportion of variance, it mainly reflected overall food intake across most food groups and was therefore less informative for interpreting packaging- or convenience-related dietary behaviours. By contrast, PC2 explained a smaller proportion of variance but captured a more specific contrast involving several food groups commonly consumed as packaged, processed, or convenience-oriented products.

Among the PCA-derived food-consumption components (Table 4), only PC2 showed a significant positive association with DPES-MX scores in the adjusted model (β = 0.81, 95% CI: 0.37 to 1.24, *p* < 0.001), whereas no significant associations were identified for PC1 or PC3. The final adjusted model showed a modest overall fit, with R^2^ = 0.217 and adjusted R^2^ = 0.147; the overall model test was statistically significant, *p* = 0.001.

**Figure 1:**
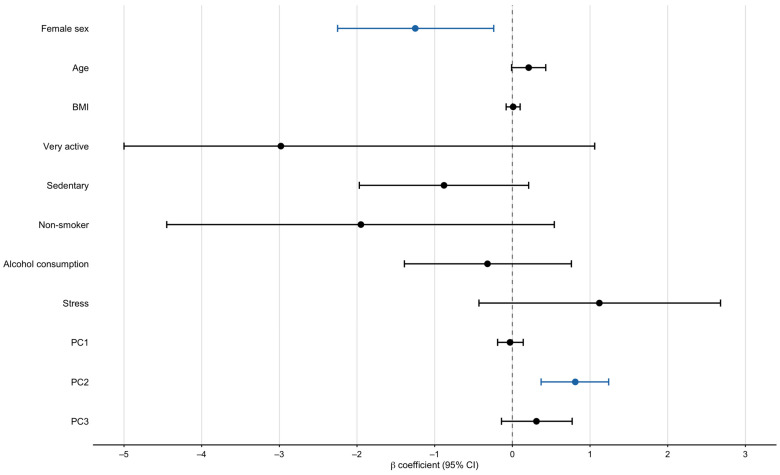
Adjusted linear regression coefficients for DPES-MX score. Points represent β coefficients and horizontal lines represent 95% confidence intervals. Blue points and lines indicate statistically significant associations (*p* < 0.05). DPES-MX, Dietary Plastic Exposure Score for Mexican Populations; BMI, body mass index; PC, principal component.

Food-group loadings for the first three principal components are presented in Table 5. The highest absolute loadings were used to support the interpretation of each component. As shown in Figure 1, higher PC2 scores were associated with higher DPES-MX scores, suggesting that this component captured a food-consumption profile with greater reliance on packaged and processed foods.

## 4. Discussion

The present study aimed to develop and preliminarily evaluate a culturally adapted dietary and behavioural score for estimating long-term exposure-related practices involving PRFC materials in Mexican populations, while also describing exposure-related dietary behaviours within a Mexican university population. Overall, the findings suggest that the DPES-MX may help identify dietary profiles linked to greater interaction with packaging-intensive food environments, particularly those characterised by sugar-sweetened beverages, processed snacks, alcohol-containing beverages, takeaway foods, and convenience-oriented consumption patterns. These findings should be considered alongside the growing literature on diet-related exposure to plastic-associated chemicals and related health outcomes. BPA, phthalates, and other food-contact chemicals have been linked in epidemiological and experimental studies to endocrine disruption, metabolic alterations, obesity, cardiovascular risk, reproductive outcomes, and neurodevelopmental endpoints [2,5,6,9]. In addition, recent evidence suggests that food-contact articles may release micro- and nanoplastics into foods during normal or intended use, although the health relevance of this route remains under active study [4]. However, the present study did not measure urinary biomarkers or microplastic burden. Therefore, the DPES-MX should not be interpreted as a direct indicator of internal exposure or disease risk, but as a preliminary tool to identify dietary and food-handling practices that may increase contact with plastic-related food-contact materials and that warrant further evaluation in biomarker-based studies.

Beyond the preliminary evaluation of the DPES-MX, the present findings provide insight into exposure-related dietary behaviours within contemporary Mexican university environments. Higher DPES-MX scores were associated with convenience-oriented dietary patterns characterised by sugar-sweetened beverages, processed snacks, sweets, alcohol-containing beverages, and packaged foods. The principal component analysis further showed that higher DPES-MX scores clustered with dietary patterns theoretically linked to repeated food-contact interactions with plastics, coatings, disposable containers, and industrial packaging systems. These findings provide indirect support for the rationale of the questionnaire and are consistent with previous exploratory approaches proposed for estimating dietary exposure to FCC in epidemiological settings where biomonitoring is not feasible [6,7,8,22]. Importantly, epidemiological studies have also reported associations between higher ultra-processed food consumption and increased urinary concentrations of phthalates and bisphenols, reinforcing the hypothesis that convenience-oriented dietary patterns may contribute to greater exposure to FCC [23,24,25]. The interpretation of the PCA results should be made with caution. The amount of variance explained by a principal component refers to variability in food-group intake, not to its association with DPES-MX scores. Therefore, a component explaining less dietary variance may still be more relevant to the DPES-MX if it captures food-consumption behaviours more closely related to packaging, convenience products, or food-contact materials. In the present study, only PC2 was significantly associated with DPES-MX scores in the adjusted model, whereas PC1 and PC3 were not. This finding supports the interpretation that a more specific convenience/packaged-food-related dietary dimension, rather than overall food intake, was associated with higher DPES-MX scores.

Women had lower DPES-MX scores and were more often classified within the low DPES-MX score category. By contrast, men reported consuming more packaged convenience products, such as instant noodles and microwaveable meals. Although the present study was not designed to identify the determinants underlying these differences, previous evidence suggests that women generally maintain greater involvement in household food preparation and exhibit higher cooking and food skills than men. Hartmann et al. [26] observed that women showed higher cooking skills across all age groups and were more frequently responsible for meal preparation within the household. They also reported that lower cooking skills were associated with greater consumption of convenience foods, including ready-to-heat and highly processed products. Jaffe and Gertler further proposed that the progressive replacement of home cooking by industrially prepared foods contributes to increased dependence on packaged and processed products within modern food systems [27].

Similarly, in Latin American populations such as Brazil, home cooking is deeply rooted in cultural and family traditions, often associated with caregiving roles and intergenerational culinary practices, and women tend to maintain higher cooking and food skills throughout adulthood [28]. Although a slight shift has occurred, with a higher percentage of men participating in cooking activities, women still make up the majority [29]. Therefore, the lower DPES-MX scores observed among women may reflect broader differences in domestic food preparation, meal planning, and lower reliance on packaging-intensive convenience foods rather than dietary choices alone.

The present findings should be interpreted as a preliminary evaluation of the DPES-MX rather than a full validation study. Importantly, the DPES-MX was designed as a formative exposure-oriented index, not as a reflective psychometric scale. In reflective scales, items are expected to represent interchangeable manifestations of a common latent construct, making internal consistency indicators such as Cronbach’s alpha central to psychometric evaluation. By contrast, formative indices combine non-interchangeable indicators that may contribute independently to the overall construct. In the DPES-MX, behaviours such as consuming canned foods, heating food in plastic containers, using plastic water dispensers, or purchasing takeaway foods in disposable packaging may reflect different exposure pathways and are not necessarily expected to be strongly correlated with one another. Therefore, limited internal consistency, if observed, would not necessarily indicate poor performance of the index, but rather the heterogeneous nature of the behaviours captured. Nevertheless, future studies should examine the behaviour of individual domains and their contribution to the total score.

The role of PCA in the present study should also be considered exploratory. The extracted components were not intended to represent predefined dietary groups, such as animal-protein or fruit-and-vegetable patterns, nor were they used as an independent validation of the DPES-MX. Instead, PCA was applied to reduce correlated food-group variables into broader dietary dimensions that could be entered into adjusted models. In this context, the association between PC2 and DPES-MX scores should be interpreted as indirect support for the conceptual coherence of the index, since this component captured a food-consumption profile partly characterised by packaged, processed, and convenience-oriented products.

The primary objective of the questionnaire was not to provide an exact quantitative estimate of internal chemical burden, but rather to identify dietary and behavioural practices linked to increased contact with PRFC materials. Similar exploratory approaches have previously been proposed for estimating dietary exposure to bisphenols and FCC in epidemiological settings where biomonitoring is not feasible [7,8]. Therefore, the DPES-MX should be considered evidence supporting the score rationale, rather than independent validation. Another important strength of the DPES-MX lies in its cultural adaptation to the contemporary Mexican food environment. Unlike generic exposure questionnaires developed in European or North American settings, the present instrument incorporated exposure-related behaviours highly prevalent in urban Mexican populations, including takeaway consumption, bottled beverage intake, packaged snack consumption, hot foods served in disposable containers, and street-food purchasing practices. These behaviours are particularly relevant because many foods sold in contemporary Mexican urban settings are commonly served in direct contact with plastic bags, foam containers, coated wrappers, or disposable packaging materials, often under high-temperature conditions. Recent studies have shown that takeaway food containers may release microplastics and plastic-associated compounds when exposed to hot food or reheating conditions, particularly in expanded polystyrene and polyethylene-based materials [30]. Consistently, a recent systematic evidence map concluded that food-contact articles can release micro- and nanoplastics during normal or intended use, although the reliability and comparability of available studies remain heterogeneous and further standardised migration testing is needed [4]. Previous studies have also demonstrated that temperature, storage duration, food composition, packaging type, lipid content, pH, and contact conditions strongly influence migration of bisphenols and other plastic-related compounds into foods [3,20,31,32,33]. Although much of the available literature has focused on BPA exposure, modern food-contact materials contain complex mixtures of plastic-associated compounds, including plasticizers, stabilisers, coatings, residual monomers, and non-intentionally added substances capable of migrating into food under real-use conditions [20]. In Mexican populations undergoing rapid nutritional transition, increasing dependence on convenience-oriented and ultra-processed foods may therefore contribute not only to poorer dietary quality but also to greater cumulative interaction with food-contact plastics [22]. This evidence supports the weighting strategy applied in the DPES-MX, where behaviours involving heating, canned foods, microwavable products, takeaway meals, and prolonged food–plastic contact received greater weighting factors due to their theoretically higher migration potential.

Some limitations should be acknowledged. First, the DPES-MX was based on self-reported dietary and behavioural information, which may be affected by recall bias and exposure misclassification. Second, the cross-sectional design does not allow causal interpretation of the observed associations. Third, the study was conducted in a relatively homogeneous university population, which may limit generalizability to other Mexican demographic groups. In addition, exposure to plastic-related compounds is multifactorial and may also derive from personal care products, indoor dust, and environmental sources beyond diet [9]. A further methodological consideration is the potential conceptual overlap between the DPES-MX and the PCA-derived dietary patterns. Although the PCA was based on food-group intake variables rather than on the packaging and food-handling items used to construct the DPES-MX, some food groups may indirectly capture convenience-oriented or packaging-intensive eating behaviours. Therefore, the association between PC2 and DPES-MX should be interpreted as evidence of conceptual coherence rather than as independent validation of the score. Finally, no biological exposure markers were available for validation, and therefore the DPES-MX should be interpreted as a preliminary exposure-oriented epidemiological tool rather than a direct estimate of internal chemical burden. Further studies are needed to evaluate the reproducibility and longitudinal stability of the DPES-MX, including test–retest reliability, correlations between individual domains and the total score, and sensitivity analyses using alternative weighting schemes. Future validation studies should also compare DPES-MX scores with urinary BPA, BPS, BPF, phthalate metabolites, or other relevant biomarkers collected in repeated samples, given the short biological half-life and high intra-individual variability of many plastic-related compounds.

## 5. Conclusions

In conclusion, the present study provides preliminary evidence that the DPES-MX could serve as a culturally adapted tool for identifying dietary and food-handling practices linked to potential exposure to PRFC materials in Mexican populations. The findings suggest that dietary patterns involving packaged beverages, takeaway products, processed snacks, and ultra-processed foods are associated with higher DPES-MX scores in Mexican university students. These associations support the rationale of the instrument, although they should not be interpreted as evidence of internal chemical exposure or health risk. The observed sex-related differences also suggest that domestic food preparation practices and cooking skills may be relevant to dietary behaviours involving food-contact materials. From a public health perspective, strengthening home-cooking skills and reducing dependence on packaged convenience foods could be explored in future studies as a plausible strategy to modify these behaviours, while also supporting healthier and less-processed dietary patterns. Future studies incorporating biological exposure markers, repeated measurements, and more diverse populations are necessary to further evaluate the epidemiological applicability of the DPES-MX and its potential utility in environmental health research.

## Figures and Tables

**Table 1:** Structure and scoring system of the DPES-MX.

Domain	Variable	Coding	Weight
Canned foods	Tuna, canned corn, canned fruit	0–3	1.00
Heating in plastic	Microwave heating in plastic containers; hot food stored in plastic while still hot	0–3	1.00
Fast-food packaging	Plastic container/bag, polystyrene/foam packaging, plastic cup	0–3	0.75
Fast-food packaging	Cardboard box, paper wrapping, aluminium wrapping, paper cup	0–3	0.50
Microwavable packaged foods	Instant noodles and microwave rice	0–3	0.50
Beverage packaging exposure	Juice in carton	0–1	0.25
	Juice in plastic container	0–1	0.50
	Water in plastic container	0–1	0.50
	Packaged beverages in plastic containers	0–1	0.50
	Packaged beverages in cans	0–1	0.75
	Plastic water dispenser use	0–3	0.50
Flexible plastic packaging	Foods consumed from flexible plastic containers or pouches	0–3	0.50
Usual food wrapping	Paper food wrapping	0–1	0.25
	Plastic food wrapping	0–1	0.75
	Aluminium food wrapping	0–1	0.50

Note: The weights represent relative theoretical weights assigned according to previous dietary BPA/plastic-exposure scoring approaches and evidence on migration potential from food-contact materials. They should not be interpreted as quantitative estimates of internal exposure or as empirically validated migration coefficients.

**Table 2:** Anthropometric and lifestyle characteristics of participants according to sex.

Variable	Total	Men	Women	Effect Size	*p*
Distribution, n (%)	152 (100.0)	85 (55.9)	67 (44.0)		
Age, years *	19.33 (2.1)	19.36 (2.4)	19.29 (1.8)	0.10	0.233
Weight, kg *	67.60 (18.4)	75.14 (20.0)	58.02 (10.0)	0.51	<0.001
Height, m *	1.66 (0.0)	1.71 (0.0)	1.59 (0.0)	0.68	<0.001
BMI, kg/m^2^ *	24.27 (5.2)	25.41 (6.0)	22.82 (3.4)	0.28	<0.001
BMI category (n, %)					
Underweight	9 (5.9)	5 (5.8)	4 (5.9)	0.21	0.071
Normal weight	92 (60.5)	44 (51.7)	48 (71.6)		
Overweight	36 (23.6)	25 (29.4)	11 (16.4)		
Obesity	15 (9.8)	11 (12.9)	4 (5.9)		
Physical Activity (n, %)					
Sedentary	100 (65.7)	58 (68.2)	42 (62.6)	0.15	0.220
Active	49 (32.2)	24 (28.2)	25 (37.3)		
Very active	3 (1.9)	3 (3.5)	0 (0.0)		
Smokers (n, %)	6 (3.95)	4 (4.7)	2 (2.9)	0.01	0.695
Alcohol consumption (n, %)	38 (25.0)	26 (30.5)	12 (17.9)	0.13	0.109

* Variables expressed as mean (SD); BMI: body mass index; effect sizes are reported as Cohen’s d for continuous variables and Cramer’s V for categorical variables.

**Table 3:** DPES-MX total and domain scores according to sex.

Domain (Mean, SD)	Total	Men	Women	Effect Size	*p*
Canned foods	1.51 ± 1.2	1.54 ± 1.3	1.48 ± 1.0	0.03	0.694
Heating in plastic	3.77 ± 1.4	3.80 ± 1.3	3.73 ± 1.4	0.03	0.757
Fast-food packaging: direct plastic contact	1.93 ± 1.4	2.03 ± 1.3	1.80 ± 1.5	0.09	0.290
Fast-food packaging: coated	1.20 ± 1.0	1.23 ± 1.0	1.16 ± 1.0	0.05	0.506
Microwavable packaged foods	0.40 ± 0.4	0.50 ± 0.5	0.27 ± 0.2	0.20	0.013
Beverage packaging	1.84 ± 0.8	1.91 ± 0.8	1.75 ± 0.8	0.10	0.217
Flexible plastic packaging	1.36 ± 0.2	1.38 ± 0.2	1.34 ± 0.3	0.06	0.457
Usual food wrapping	0.59 ± 0.2	0.59 ± 0.2	0.60 ± 0.2	0.03	0.704
Total DPES-MX score	12.60 ± 2.9	12.98 ± 2.9	12.12 ± 2.9	0.14	0.089
DPES-MX score category (n, %)					
Low	51 (33.5)	22 (25.8)	29 (41.7)	0.17	0.037
Moderate	51 (33.5)	32 (37.6)	19 (26.8)	0.08	0.302
High	50 (32.8)	31 (34.1)	19 (31.3)	0.07	0.377

Values are expressed as mean (SD) unless otherwise indicated. DPES-MX, Dietary Plastic Exposure Score for Mexican Populations; SD, standard deviation. Effect sizes are reported as Cohen’s d for continuous variables and Cramer’s V for categorical variables. DPES-MX score categories were defined according to tertiles of total DPES-MX score distribution in the overall sample. Percentages within sex-specific columns are calculated.

**Table 4:** Adjusted linear model for DPES-MX including food-pattern PCA scores.

Variable	Beta	95% CI	*p*
Women	−1.06	−2.25 to −0.05	0.016
Age	0.21	−0.01 to 0.43	0.058
BMI	0.01	−0.08 to 0.10	0.753
Very active	−2.98	−7.02 to 1.06	0.222
Sedentary	−0.88	−1.97 to 0.21	0.112
Non-smoker	−1.95	−4.45 to 0.54	0.124
Alcohol consumption	−0.32	−1.39 to 0.76	0.564
Self-perceived stress	1.12	−0.43 to 2.68	0.156
PC1	−0.03	−0.19 to 0.14	0.763
PC2	0.81	0.37 to 1.24	<0.001
PC3	0.31	−0.14 to 0.77	0.177

BMI, body mass index; PCA, principal component analysis; PC, principal component. β values represent adjusted linear regression coefficients. Reference categories were men for sex, active participants for physical activity, smokers for smoking status, non-drinkers for alcohol consumption, and participants without self-perceived stress for stress.

**Table 5:** Food-group loadings for the first three principal components.

Food Group	PC1	PC2	PC3
Dairy products, g/day	−0.21	−0.13	**−0.48**
Red meat, g/day	−0.24	0.04	**−0.31**
Poultry, g/day	−0.22	**−0.35**	**−0.44**
Fish and seafood, g/day	−0.19	**0.44**	−0.25
Vegetables, g/day	−0.27	−0.11	0.24
Fruits, g/day	−0.28	0.04	−0.02
Legumes and soy, g/day	−0.25	0.05	**0.33**
Tubers, g/day	−0.27	−0.15	0.25
Cereals and grain-based foods, g/day	−0.27	−0.26	0.04
Fats and oils, g/day	−0.26	−0.11	−0.02
Sugars and sweets, g/day	−0.25	0.27	0.03
Cookies and snacks, g/day	−0.24	0.18	0.04
Sugar-sweetened beverages, g/day	−0.21	0.15	0.14
Sugar-free beverages, g/day	−0.29	−0.10	−0.06
Alcoholic beverages, g/day	−0.17	**0.59**	0.02
Nuts, g/day	−0.19	−0.25	**0.39**
Sauces and condiments, g/day	−0.26	−0.03	0.02

PCA, principal component analysis; PC, principal component. Loadings ≥ |0.30| are shown in bold. The sign of PCA loadings is arbitrary; interpretation was based on the relative contribution of food groups within each component.

## Data Availability

There are restrictions on the availability of data for this study due to the signed consent agreements around data sharing, which only allow access to external researchers for studies following the project’s purposes. Requestors wishing to access the data used in this study can make a request to mariscal@ugr.es.
